# Common lizard microhabitat selection varies by sex, parity mode, and colouration

**DOI:** 10.1186/s12862-023-02158-2

**Published:** 2023-09-04

**Authors:** Hans Recknagel, William T. Harvey, Megan Layton, Kathryn R. Elmer

**Affiliations:** 1 School of Biodiversity, One Health & Veterinary Medicine, College of Medical, Veterinary & Life Sciences, University of Glasgow, Glasgow, G12 8QQ UK; 2 Present Address: Biotechnical Faculty, Department of Biology, University of Ljubljana, 1000 Ljubljana, Slovenia; 3 Present Address: Roslin Institute, University of Edinburgh, Edinburgh, UK

**Keywords:** Dorsal colour, Pattern, Crypsis, Viviparous, Oviparous, Microhabitat, Basking, Squamate, Environment

## Abstract

**Background:**

Animals select and interact with their environment in various ways, including to ensure their physiology is at its optimal capacity, access to prey is possible, and predators can be avoided. Often conflicting, the balance of choices made may vary depending on an individual’s life-history and condition. The common lizard (*Zootoca vivipara*) has egg-laying and live-bearing lineages and displays a variety of dorsal patterns and colouration. How colouration and reproductive mode affect habitat selection decisions on the landscape is not known. In this study, we first tested if co-occurring male and female viviparous and oviparous common lizards differ in their microhabitat selection. Second, we tested if the dorsal colouration of an individual lizard matched its basking site choice within the microhabitat where it was encountered, which could be related to camouflage and crypsis.

**Results:**

We found that site use differed from the habitat otherwise available, suggesting lizards actively choose the composition and structure of their microhabitat. Females were found in areas with more wood and less bare ground compared to males; we speculate that this may be for better camouflage and reducing predation risk during pregnancy, when females are less mobile. Microhabitat use also differed by parity mode: viviparous lizards were found in areas with more density of flowering plants, while oviparous lizards were found in areas that were wetter and had more moss. This may relate to differing habitat preferences of viviparous vs. oviparous for clutch lay sites. We found that an individual’s dorsal colouration matched that of the substrate of its basking site. This could indicate that individuals may choose their basking site to optimise camouflage within microhabitat. Further, all individuals were found basking in areas close to cover, which we expect could be used to escape predation.

**Conclusions:**

Our study suggests that common lizards may actively choose their microhabitat and basking site, balancing physiological requirements, escape response and camouflage as a tactic for predator avoidance. This varies for parity modes, sexes, and dorsal colourations, suggesting that individual optimisation strategies are influenced by inter-individual variation within populations as well as determined by evolutionary differences associated with life history.

**Supplementary Information:**

The online version contains supplementary material available at 10.1186/s12862-023-02158-2.

## Background

Within their environments, individuals of any animal species are presented with many options for where to spend their time. These microhabitat selection decisions mainly depend on the trade-offs between finding food, engaging in social interactions, and avoiding predators [1–4]. Among these, predator avoidance may play the most important role, as an incautious selection can lead to immediate death [3]. To avoid predation, an individual is usually faced with two choices: hiding or fleeing. Camouflage, the similarity between the colouration and pattern of an animal and its surroundings, is a striking example of adaptation that has become a model system within evolutionary research [5–9]. Studies on camouflage in wild animals suggest that prey animals inhabiting a variety of coloured microhabitats will either compromise between different microhabitats, or specialise in one microhabitat to optimise predator avoidance [7, 10]. For example, bark-resting moths such as the peppered moth (*Biston betularia*) show a preference for microhabitats with a similar colouration to themselves [11, 12] and will even reposition their body to enhance camouflage [13–15]. Camouflage may also depend on the variety of different microhabitats encountered by an individual. For example, desert lizards (*Sceloporus magister*) have compromised on a coloration that is intermediate between two or possibly more of the microhabitats that they use [7].

In squamates, dorsal patterning and colouration has mainly evolved to match the surrounding substrate for crypsis [16–18]. However, colour and patterning may also have evolved as a response to sexual signalling and environmental pressures, including temperature and protection from ultra-violet radiation [19–24]. For example, in the viper *Montivipera raddei* species complex, dorsal pattern colouration varies in association with thermal factors, including temperature and solar radiation [25]. However, predator avoidance tactics might change during an individual’s lifetime, depending on ontogenetic changes in colouration, sprint speed (escape performance), or changes in behaviour due to reproduction and mate acquisition [26–28]. Overall, studies find a complex relationship between colouration, patterning, sex, and animal personalities, suggesting that there are interdependent trade-offs in the evolution of this variation [23, 29, 30].

Providing opportunities for camouflage is an important consideration for habitat selection, but microhabitats critically also offer protection from predators [31, 32], opportunities for encountering prey [4, 33], or thermoregulatory benefits for ectotherms [2, 34]. For example, vegetation cover and short flee distance to hiding spots allow animals to avoid detection and decrease predation risk [32, 35]. On the other hand, open basking sites provide optimal opportunities to maximise exposure to sunlight and thus have thermoregulatory benefits for ectotherms [36, 37], but are more exposed to potential predators [38]. Therefore, individuals need to carefully choose the amount of time they spend in different types of microhabitat. In addition, these choices may depend on the sex of an individual and the season. For example, females are usually less mobile during pregnancy, and therefore need to adjust their behaviour as their flight speed reduces [39, 40].

While it has been well demonstrated that lizards select microhabitats with particular characteristics (e.g., for thermoregulation [41–44] or predator avoidance [1, 45]), few studies have tested whether individuals within the same species that show intraspecific variation in appearance actively choose specific backgrounds within a microhabitat. Recent studies indeed suggest that, in some species, individuals may actively choose a habitat that matches their own colouration. For example, in ground-nesting birds, individuals match their own colouration and their eggs to the habitat they nest in [46] and escape more readily when their own match to the background – but not their clutch’s – is poor [47]. In Aegean wall lizards (*Podarcis erhardii*) inhabiting Greek islands, individuals match their own colouration to the background, with the effect being stronger in females and in areas with higher predation risk [15]. Therefore individual decision-making on site use likely involves the local environment availability and an organism’s colour variation.

The common lizard (*Zootoca vivipara*) is a small, ground-dwelling lizard found across Eurasia. In addition to having the largest distributional range of any terrestrial reptile, it shows striking variation in reproductive aspects of life-history: some populations are egg-laying (oviparous), while others are live-bearing (viviparous) [48, 49]. Common lizard reproductive season starts in early spring and ends in summer, and during this time gestating females may double in weight [50]. This period is also when common lizards are most vulnerable to predators that often rely on visual cues, such as birds of prey, snakes and some mammals.

Common lizards show considerable variation in inter-individual colouration and dorsal patterning [51], with reticulated or linear patterns of dark on lighter brown background, or an intermediate pattern between reticulated and linear [23]. An individual’s dorsal patterning is established during the first year and is then stable throughout its lifetime [23]. Common lizards do not have active colour change to match or camouflage against particular substrates, as known in other lizards such as chameleons [52]. Features such as body condition and heterozygosity play important roles in common lizard mate choice [53, 54] but dorsal colour or pattern do not seem to be crucial cues. Instead the frequencies at which reticulated and linear forms occur in a population vary depending on environmental conditions and on genetic inheritance [23]. However, the association of dorsal colour and pattern on the landscape has not been investigated in detail either for males or females.

In this study we assessed microhabitat selection in syntopically occurring oviparous and viviparous common lizards during the reproductive season. We first tested if common lizard microhabitat selection was specific in the context of the environment available to them. In addition, we checked if their choice was associated with parity mode (oviparous or viviparous) and sex. Finally, we tested if common lizard dorsal colouration and patterning was associated with colouration of the basking site while accounting for size and sex differences. The hypotheses we tested were: (i) particular features of microhabitat are preferred by common lizards, (ii) oviparous and viviparous common lizards differ in microhabitat selection, (iii) pregnant females are encountered in a different microhabitat than males, (iv) dorsal colouration matches the colouration of the lizard’s basking site, and (v) dorsal patterning is associated with basking site colouration.

## Results

### Lizard selected microhabitat differs between sexes and parity mode

**Fig. 1:**
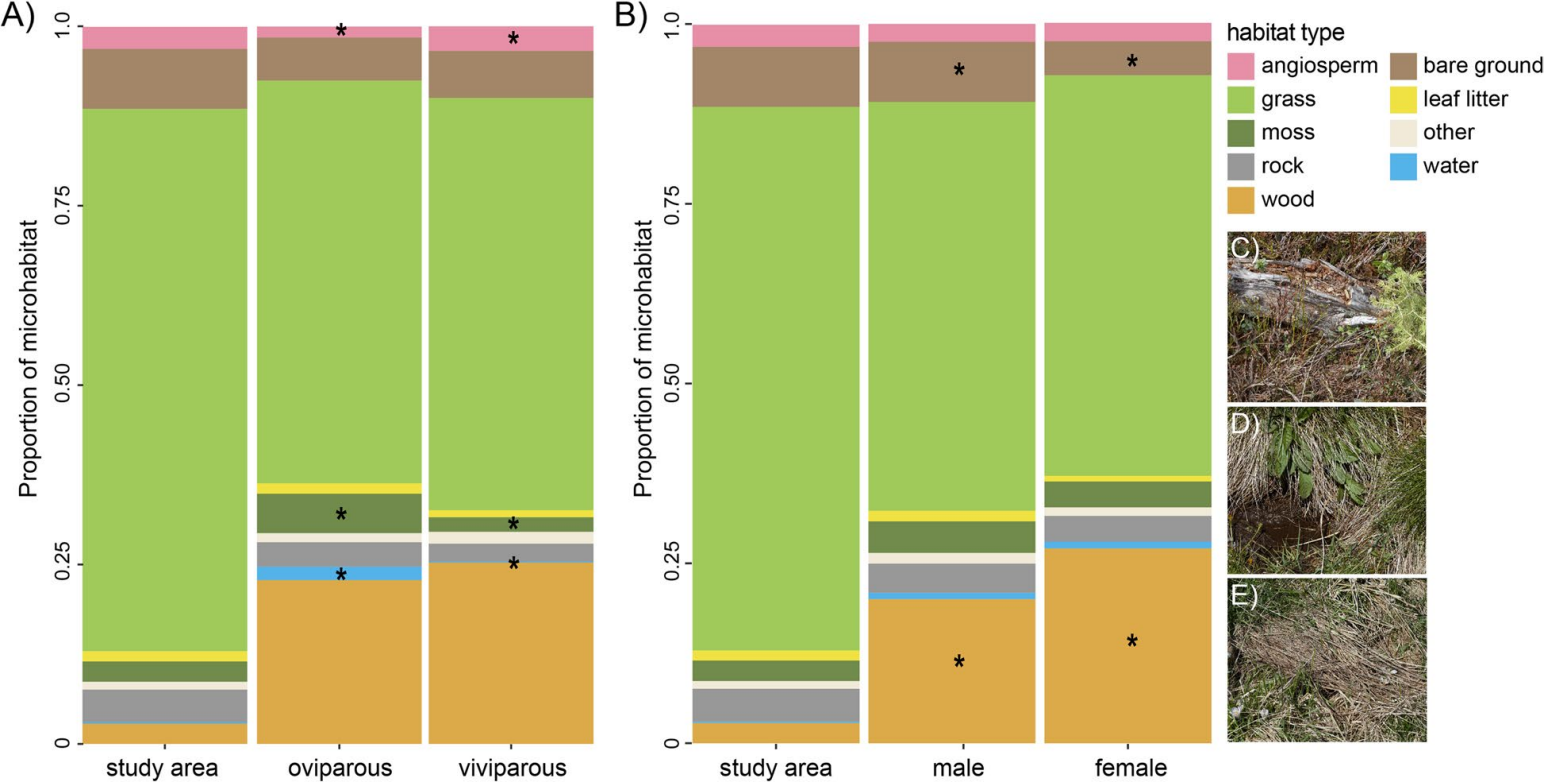
Relative proportions of habitat types encountered overall in the area (‘study area’) and within the microhabitats chosen by (**A**) oviparous and viviparous and (**B**) male and female lizards. Habitat types that differed between parity modes (**A**) or sex (**B**) are marked by an asterisk. Examples of different habitats are shown in panels **C** - **E**), including typical basking sites for common lizards, such as **C**) a dried log among heather, **D**) a dried clump of grass next to water, and **E**) patches of dry grass in the meadow

**Fig. 2:**
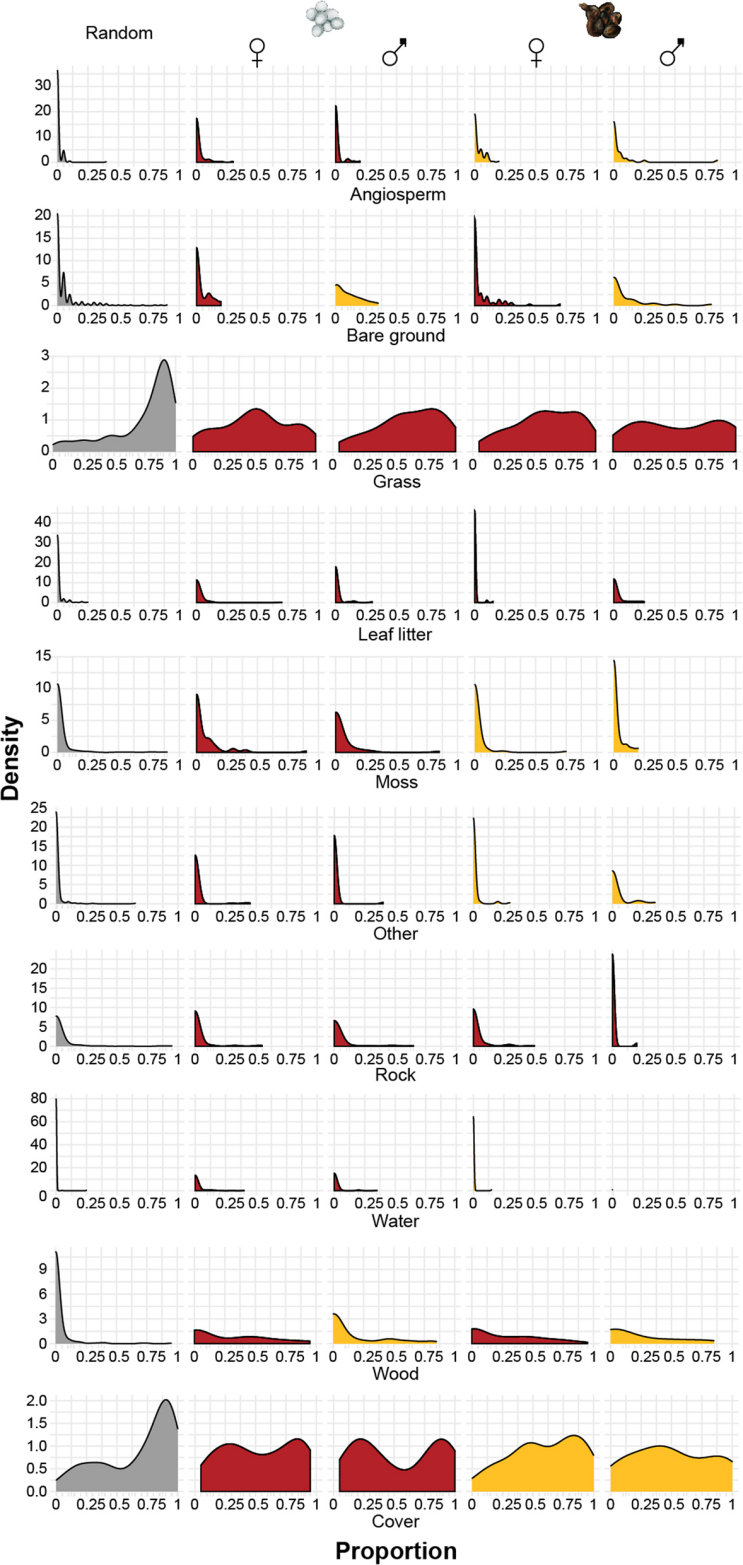
Proportion of habitat component and factors that performed best (as assessed by DIC) in explaining variance in each habitat component. Different colours in the kernel density plot represent factors that differed significantly in the best performing model. The habitat used by lizards differed in all its components from the study area available (‘Random’; grey colour). The combination of red and yellow coded colours indicated that either parity mode or sex was another significant factor. For example, in cases where parity mode was a significant factor, the second and third column (oviparous males and females; coded red) differed from the fourth and fifth column (viviparous males and females; coded yellow)

Common lizards apparently chose specific microhabitats within the sampled study area. This is shown by significant differences between the relative proportions of microhabitat categories available in the area and the microhabitat used by the lizards (Figs. 1 and 2). This was exemplified by DIC (deviance information criterion) estimates for the preferred simple lizard model (where common lizards are assumed to be a homogenous group, Table 1, rows 2 and 3) being at least 5 lower than the null model, which assumed no difference between sites with common lizards and random sites for all nine habitat types modelled (Table 1, row 1). In particular, common lizards tended to be present in areas with higher proportions of angiosperms, leaf litter, moss, water and wood, and lower proportions of cover, rocks, grass and bare ground than were randomly available in their surroundings (Table 1; Additional file 1 (Table S1); Fig. 2). Where a simple model distinguishing between lizards and randomly available sites was preferred, common lizards were present in areas with a lower proportion of grass (${\mu }_{lizard}=0.55$ and ${\mu }_{random}=0.70$) and higher proportions of leaf litter (${\mu }_{lizard}=0.18$ and ${\mu }_{random}=0.10$) than randomly available. When present, rock was found in similar proportions across groups (${\mu }_{lizard}=0.27$ and ${\mu }_{random}=0.29$), but was more likely to be completely absent in the vicinity of lizards (${\alpha }_{lizard}=0.89$ and ${\alpha }_{random}=0.83$).

**Table 1:** Deviance information criterion (DIC) for zero-and-one-inflated beta (ZOIB) regression models explaining the variation in the proportion of microhabitat types in the immediate proximity of lizards and in randomly selected quadrat squares

Model^1^	Precision^2^	Habitat type								
	**scheme**	Angiosperm	Grass	Leaf litter	Moss	Rock	Water	Wood	Cover	Bare ground
Null	shared	49.7	111.1	193.8	352.6	352.0	111.2	592.4	198.6	352.8
Lizard	shared	-17.1	**72.1**	190.7	346.9	352.4	105.6	479.2	184.8	354.5
Lizard	variable	-39.4	74.7	**186.2**	346.3	**345.1**	112.5	481.2	187.1	344.7
Sex	shared	-16.4	75.5	193.5	350.6	356.5	110.2	**477.0**	188.0	350.1
Sex	variable	-37.5	76.9	187.0	350.7	348.0	118.5	480.5	190.5	**339.3**
parity mode	shared	-32.0	73.5	194.6	**341.7**	356.2	**101.6**	479.9	**175.3**	355.6
parity mode	variable	**-54.0**^3^	75.1	189.1	342.0	347.8	168.5	482.1	177.2	345.2
parity * sex	shared	-32.4	72.9	194.4	342.4	356.7	102.9	479.7	175.8	355.2
parity * sex	variable	-53.0	75.5	189.0	341.9	347.9	110.2	482.3	177.4	345.7

^1^The null model assumes no difference in microhabitat proportion in lizard and randomised locations. The lizard model allows for variation between lizard and randomised locations. Further models allowed proportions for lizards of different sex, parity mode, or both (parity * sex) to be drawn from different underlying distributions

^2^The precision parameters in the beta component of the ZOIB distribution was either shared by all observations or allowed to vary between lizard and randomised observations

^3^For each microhabitat, preferred models selected according to DIC are indicated by bold text and shaded cells

For six of the nine habitat types, more complex models based on differences between lizards were preferred (Table 1). Males lizards were more likely to be observed with no bare ground (proportion = 0) in the vicinity (${\alpha }_{random}=0.67$) compared with both females and random availability (${\alpha }_{random}=0.58$ and ${\alpha }_{female}=0.57$) (Additional file 1 (Table S1). When present, the proportion of wood in the vicinity of females of both parity modes was higher (${\mu }_{female}=0.47)$ compared with male lizards (${\mu }_{female}=0.42)$ or random (${\mu }_{female}=0.22$). Viviparous common lizards were found more often in association with angiosperms (${\alpha }_{viviparous}=0.65$) compared with oviparous lizards (${\alpha }_{oviparous}=0.87$). Oviparous lizards were less likely to be found in the absence of water (${\alpha }_{oviparous}=0.91$) than viviparous lizards or randomly available (${\alpha }_{viviparous}=0.99$ and ${\alpha }_{random}=0.99$) and when present, water made up a higher proportion of an oviparous lizard’s surroundings (${\mu }_{oviparous}=0.19$ while ${\mu }_{viviparous}$ and ${\mu }_{random}=0.14$). Oviparous common lizards were also less likely to be found in the absence of moss (${\alpha }_{oviparous}=0.71$) than viviparous lizards or randomly available (${\alpha }_{viviparous}=0.86$ and ${\alpha }_{random}=0.88$). Finally, viviparous lizards were more likely to have a total presence or absence of cover (${\alpha }_{viviparous}=0.12)$ with a proportion of 1 more likely than absence (${\gamma }_{viviparous}=0.59$), meanwhile oviparous common lizards exhibited a bimodal distribution in their preference of how much cover was available in their habitat, while viviparous had a more unimodal distribution (Fig. 2).

### Dorsal colouration and patterning

Dorsal colouration was associated with parity mode, sex, body length, and body weight (Table 2). More specifically, viviparous and larger individuals tended to have a higher hue (Table 2; Additional file 2 (Table S2); Additional file 3 (Fig. S1)). Moreover, viviparous, male, larger, and lighter-weight lizards tended to have a more intense colour (model 1 (saturation); Table 2; Additional file 2 (Table S2)). Finally, oviparous had a lighter dorsal colouration compared to viviparous individuals (estimate = -3.12, *t*_*1,250*_ = -3.34, *P* < 0.001; Table 2; Additional file 2 (Table S2)).

**Table 2:** Association between lizard dorsal colouration, patterning, microhabitat and basking site colouration. Shown are all statistical models that showed a significant association with the response variable. Abbreviations: Num. = numerator; Den. = denominator; df = degrees of freedom

Model ID	Response variable	Explanatory variable	Test	Num. df	Den. df	t - or F - value	*p* - value	
1	dorsal colouration (H)	body length	GLM	3	249	3.31	< 0.01	**
		parity mode				3.57	< 0.001	***
	dorsal colouration (S)	sex	GLM	5	247	2.90	< 0.01	**
		body length				3.30	< 0.01	**
		parity mode				2.01	< 0.05	*
		body mass				-3.72	< 0.001	***
	dorsal colouration (L)	parity mode	GLM	2	250	-3.34	< 0.001	***
2	dorsal patterning	sex	GLM	3	387	3.05	< 0.01	**
		body length				2.79	< 0.01	**
3	dorsal colouration (H)	dorsal patterning	ANOVA	2	299	3.04	< 0.05	*
	dorsal colouration (L)	dorsal patterning	ANOVA	2	299	6.08	< 0.01	**
4	basking site colouration (H)	habitat colouration (H)	LM	2	155	8.75	< 0.001	***
	basking site colouration (S)	habitat colouration (S)	LM	2	155	11.35	< 0.001	***
	basking site colouration (L)	habitat colouration (L)	LM	2	155	11.51	< 0.001	***
5	dorsal colouration (H)	basking site colouration (H)	LM	2	160	3.01	< 0.01	**
	dorsal colouration (L)	basking site colouration (L)	LM	2	160	-2.14	< 0.05	*
6	dorsal colouration (H)	habitat colouration (H)	LM	2	157	5.29	< 0.001	***

**Fig. 3:**
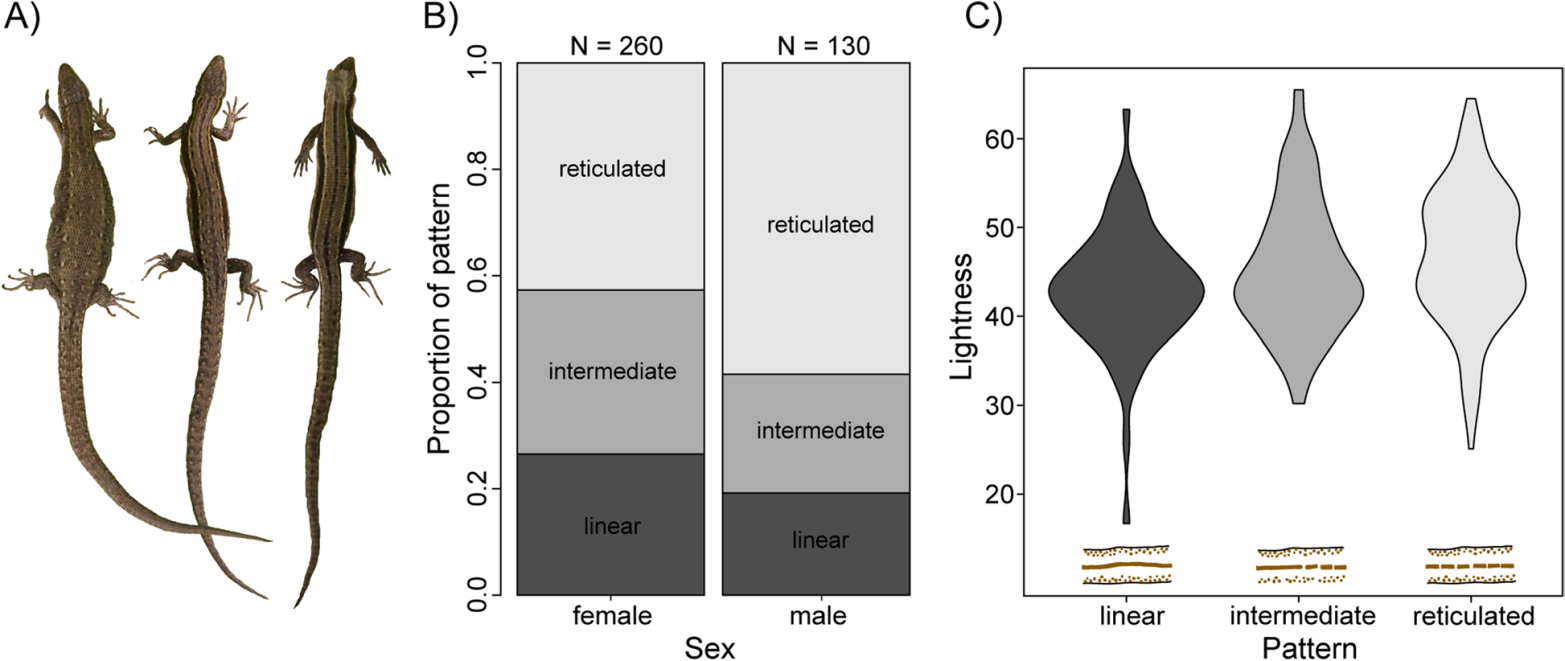
Dorsal pattern differs between sex and is associated with dorsal colouration. **A** Three females with examples of a reticulated, intermediate and linear pattern (left to right). **B** Proportions of individuals of reticulated, intermediate, or linear dorsal patterning, shown for males and females separately. **C** Reticulated individuals tend to have an overall lower lightness score in dorsal colouration

Dorsal patterning was associated with body length and sex (model 2; Table 2; Fig. 3; Additional file 2 (Table S2)). Specifically, a larger proportion of males had a reticulate pattern compared to females (estimate = 1.068, *z*_1,387_ = 3.05, *P* < 0.01; Table 2; Fig. 3B) and smaller lizards were more likely to have a linear pattern (estimate = 0.071, *z*_1,387_ = 2.79, *P* < 0.01; Additional file 2 (Table S2)). Individuals with a reticulate pattern generally had a lighter dorsal colouration (model 3; *F*_2,299_ = 3.28, *R*^2^ = 0.015, *P* < 0.05; Fig. 3C; Additional file 2 (Table S2)).

### Common lizard colour matches microhabitat and basking site

Common lizard dorsal hue ranged from 27 to 51 (mean: 36.8), basking sites from 20 to 69 (mean: 47.3) and habitat from 33 to 69 (mean: 54.9). Hue was positively correlated between habitat and basking site (model 4; estimate = 0.39, *t*_1,160_ = 8.75, *R*^2^ = 0.331, *P* < 0.001;) and more variable in basking sites than habitat (standard deviation: 10.6 vs. 7.2). Common lizard lightness ranged from 25.1 to 64.9, basking site from 12.9 to 79.4, and overall microhabitat from 17.3 to 47.6 (Additional file 4 (Fig. S2)). Basking site lightness and overall habitat lightness were significantly correlated (model 5; estimate = 0.33, *t*_1,160_ = 10.81, *R*^2^ = 0.427, *P* < 0.001; Table 2; Additional file 2 (Table S2)), but the distributions differ, with basking sites being lighter (mean: 36.8 vs. 30.1) and more variable in lightness than habitat (standard deviation: 12.7 vs. 6.6; Additional file 4 (Fig. S2)).

**Fig. 4:**
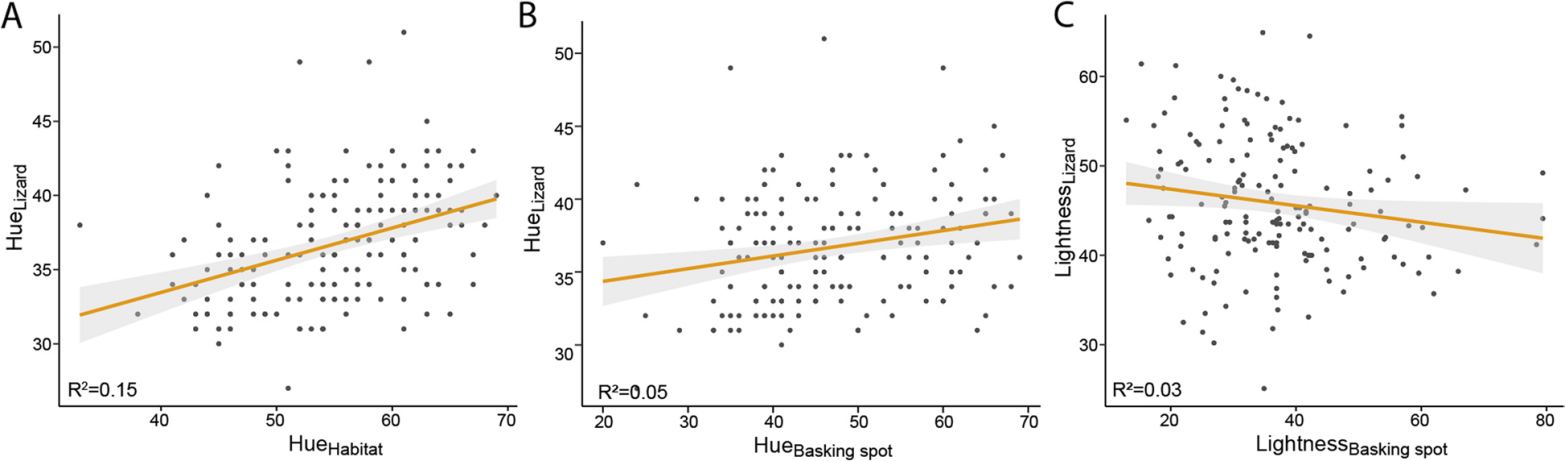
Positive relationship between common lizard dorsal colouration and the colour of its local habitat. The plots show lizard hue compared to the hue of (**A**) its habitat and (**B**) its basking site. Plot (**C**) illustrates the relationship between lizard lightness and the lightness of its surrounding basking site. Effect sizes are included as R^*2*^ derived from linear models

Dorsal colouration was associated with the colour of the basking microhabitat selected by the lizard (model 6). Specifically, the hue of the lizard’s dorsal area correlated with the hue of the habitat (estimate = 0.21, *t*_1,162_ = 5.29, *R*^2^ = 0.151, *P* < 0.001; Table 2; Fig. 4) and the basking site (estimate = 0.09, *t*_1,162_ = 3.00, *R*^2^ = 0.05, *P* < 0.01; Table 2; Fig. 4). Therefore, lizards generally tended to prefer habitats and basking sites that matched their own colouration. Moreover, lizards tended to choose basking sites that were slightly darker than their own colouration (estimate = -0.10, *t*_1,162_ = -2.14, *R*^2^ = 0.03, *P* < 0.05; Table 2; Fig. 4). In contrast to colouration, dorsal patterning, i.e. being linear or reticulated, was not associated with basking site or habitat colouration (N = 161, *P* > 0.1; Additional file 2 (Table S2)).

## Discussion

Our findings suggest that common lizards in their natural environment actively select their microhabitat, presumably to optimise thermoregulatory and physiological requirements while minimising risk. Lizards chose all habitat components in markedly different proportions than what was available to them in the immediate area. We showed that common lizards generally avoid bare rocks and bare ground. In this way, we suggest they can avoid detection and the risk of predation in such exposed habitats, an important consideration given that they are rather slow, ground-dwelling lizards. Common lizards have relatively high humidity requirements for their environment [55, 56], and this was reflected in their preference for a wetter habitat with more moss. Moreover, leaf litter and angiosperms were preferred, presumably providing options for escaping while being less visible to predators.

As basking sites, common lizards often use wood, which was also enriched in their microhabitat relative to the immediate area. Wood generally matches well with the lizard’s background colouration and also offers crevices for quick access to escape refuges [27]. In addition, wood heats up quickly and therefore provides optimal options for thermoregulation [57]. Microhabitats with less vegetation cover available than what is randomly distributed in the environment were also preferred, presumably because common lizards require basking sites and taller vegetation reduces the amount of solar radiation that can be used for thermoregulation.

In addition to these general preferences, microhabitat selection depended on an individual’s life history: site selection differed between males and females, and oviparous and viviparous lizards. Females preferred habitats with good basking options (more wood) and less open space (‘bare ground’). During the reproductive season, females are pregnant and reduced in speed [40, 58, 59], and therefore may require relatively ‘safer’ microhabitats where they can attain their optimal body temperature quickly but also escape to a hiding spot within short distances. By staying in close proximity to a refuge and exhibiting a low flight tolerance [27], female common lizards may offset their reduced speed during pregnancy to avoid predation. By performing running performance tests in addition to microhabitat choice experiments, this potential sex-bias could be tested in the future using a larger and more balanced sample size.

Parity mode was also associated with common lizard microhabitat selection. Specifically, oviparous common lizards were found in wetter microhabitats with a higher proportion of moss and a lower proportion of flowering plants. This confirms a previous study which found that oviparous common lizards are found in wetter habitats [60]. We propose that the reason for this may be that for optimal development, egg clutches need a humid environment. Egg clutches of oviparous common lizards have been found below moss and within humid dead wood (personal observation), and in captivity egg clutches are usually deposited below moisturised moss when given the option [50]. Viviparous common lizards were found in microhabitats with a larger proportion of angiosperms, while other components did not differ between parity modes. In summary, we show that an individual’s sex and life history (male vs. female, oviparous vs. viviparous) are associated with microhabitat choice in common lizards in their natural environment, presumably to match physiological requirements and trade-offs with predation risk. This suggests long-term evolutionary differences play out in the behavioural strategies of individuals on the landscape.

Our findings further suggest that common lizards select particular microhabitats and basking sites compared to the background matrix and that this is associated with their dorsal colour. Individuals tended to choose a habitat and basking site colour closer to their own colouration (measured as hue) than other available options. This result is in agreement with other studies suggesting lizards match background to their own colouration in order to reduce detection by predators [15, 61, 62]. However, in respect to avian predators our result should be interpreted cautiously, as we did not measure the full spectrum of the lizard and its background. Both lizards and birds cover UV light in their visual spectra, which we did not measure here. In contrast to the positive correlation between the lizard’s hue and its habitat, the lightness of the lizard’s basking site tended to be darker than its own colouration. A potential explanation could be that lizards optimise their thermal environment during basking. As darker surfaces allow for more efficient thermoregulation, lighter individuals, which presumably require more time to heat up compared to darker individuals [63], may offset this difference by choosing darker surfaces while basking. In summary, our results suggest that lizard individuals are faced with a trade-off between heating efficiently and matching their background.

How common lizards choose their habitat and basking site in relation to their own colouration is an intriguing question. It is possible that lizards can visually assess their own dorsal colouration by curling, a behaviour they typically also adopt while basking. Alternatively, the locus or loci for dorsal colouration may be genetically linked to behavioural preferences for habitats that best match their colour and pattern. Another alternative may be that lizards learn to optimise their camouflaging behaviour from previous encounters with predators [64]. Basking sites differed from the otherwise immediate microhabitat in colouration and in composition of substrate and vegetation, strongly suggesting they are actively selected relative to the immediately available microhabitat.

Our findings also demonstrate the variation in common lizard colouration among individuals. In particular, colour intensity differed significantly between individuals of different parity modes, sex, body length and body weight. Oviparous individuals differed from viviparous individuals in all three dorsal colour variables: viviparous individuals had larger values in hue and saturation, and a lower value in lightness. While these differences were subtle, it confirms impressions from the field that oviparous individuals tended to be slightly lighter than viviparous. Moreover, the hue was shifted towards larger values in larger individuals, visible as a change from a darker brown towards a lighter brown. This is consistent with common lizards undergoing colour changes during ontogeny, while the correlation with body weight and overall fitness warrants further investigation. Reticulate-patterned individuals tended to be lighter in dorsal colouration. We also found that pattern is significantly associated with sex, with reticulated patterns being more common in males and linear patterns being more common in females. That dorsal patterning differs between sexes is in agreement with another study showing a higher proportion of linear patterns in females in wall lizard (*Podarcis muralis*) populations [24] and in a different common lizard lineage [23]. Previous research comparing dorsal patterns of common lizards and exploratory behaviour found linear individuals seem to be bolder in transitioning between habitat patches without cover, and have higher immigration rates compared to reticulate individuals [23, 29]. Therefore, there is the potential that complex behavioural traits are associated with dorsal patterning, though here we did not find this to be associated with different basking site selection.

As dorsal patterning did not have a significant effect on microhabitat selection, we suggest that the relatively darker linear morph being more abundant in females might be a thermoregulatory adaptation. A darker linear patterning may warm up the ventral column quicker during basking and improve thermoregulation [23, 24]. A higher frequency of the linear pattern in females might be explained by an increase in energy from thermoregulation aiding to escape from predators whilst gravid [24, 65, 66]. In general, the grain and geometry of a pattern, together with body colouration, may represent random samples of those visual features of the background and aid in camouflage whilst basking [61, 67]. Patterning may also cause disruptive camouflage by breaking body lines or merging body margins to match with the substrate [61, 68–70]. Although we did not find an association between dorsal patterning and basking site in common lizards at this relatively small spatial scale, patterning might be related to habitat on a larger scale, for example when habitats differ more substantially from each other (e.g., alpine meadows compared to peatland meadows).

## Conclusions

Overall, our key findings suggest that common lizards selectively choose their microhabitat and that this is non-random relative to the immediately available sites, presumably to balance life-history dependent physiological requirements, camouflage, and predator avoidance. Common lizards are preferentially found in backgrounds that enhance matching, potentially for camouflage against predators in their natural microhabitats whilst staying close to cover to enable quick escape. Further work is needed to understand the active choice in these site selections by lizards and whether camouflaged background matching benefits survival. Other studies have suggested that genetic control alongside visual input may underlie adaptive background choices, and individuals may learn to discriminate between camouflaging and non-camouflaging backgrounds [15]. Although this study tests true individual colours (as captured by camera RGB values) and does not rely on colours being indicated by the human visual system, other studies have relied on human-oriented indices [71–73]. Measures of crypsis based on the human visual system may be biased or differ from that of other animals, as different groups of animals can vary greatly in their visual systems [74–77]. Finally, future studies of reptile crypsis will benefit from using spectral reflectance data covering a wider spectrum than digital photography, and linking the background and prey colouration directly to the visual system of potential predator(s) [15, 61, 78–80].

## Materials and methods

### Study area and sampling

The study was carried out in the Carinthian Alps in Austria in the Gailtal valley. The study area covers six sites that are separated by visible barriers (e.g. rivers, roads, forests) and a total area size of approximately 0.3 km^2^, with an altitude range from 1350 to 1550 m. The area is used for grazing cows and goats during summer months (June-September) and the habitat is dominated by alpine meadow. The study area is one of the few contact zones between egg-laying and live-bearing common lizards, two genetically differentiated and distinct phylogenetic lineages [48, 49, 60].

A total of 394 individuals were caught by hand from May to July 2016 and sexed by the presence of a penile bulge in males and its absence in females. Individuals were photographed under standardised conditions to quantify colour information accurately (see next section). Snout to vent lengths (SVL) and tail lengths (TL) were measured using digital callipers (Moore and Wright) and weights were measured using a Pesola Micro-Line Spring Scale. All female lizards were kept in individual plastic terraria (28 × 19 × 14 cm), provided with hiding places, wet moss, water and food (mealworms, crickets) ad libitum [50]. Following laying eggs or giving birth, a tail clip from each female was taken for genetic analyses and she was then released at the point of capture. Live-born young were released with their mother, while egg clutches were incubated under 24 °C and released at the point of their mother’s capture upon hatching. Parity mode was assessed by phenotype (i.e., if individuals laid a clutch of eggs or gave birth to living young) and SNP genotyping [49]. For genotyping, ddRADSeq libraries were constructed and sequenced on an Illumina HiSeq 4000 machine at 2 × 150 bp read length. Individuals were demultiplexed using STACKS [81] and mapped to the common lizard reference genome [82]. SNPs were filtered and extracted by a required presence of > 50% across individuals and a minimum allele frequency of 10%. An ADMIXTURE analysis was run on all individuals using K = 2 to account for the oviparous and viviparous phylogenetic lineages encountered in the area. If the membership value (Q) fell between 0.1 and 0.9, individuals were recorded as admixed and excluded. Higher or lower values were recorded as purely oviparous or viviparous, respectively.

### Colouration and pattern data generation

Images were taken in RAW format to preserve colour information under standard settings (fixed ISO: 160, aperture: 8, exposure compensation: 0) with a Canon 70D and a 60 mm fixed lens. Each image included an ‘X-rite ColorChecker’ board to standardise colouration between images and capture accurate colour [83, 84]. First, a Digital Negative (DNG) copy of each image was made and then run through the colour checker software (X-rite ColorChecker) which establishes an accurate colour foundation in form of a profile. Next, this profile is applied to the original image before analysing to ensure standard and accurate colour results. Images were processed using Adobe Photoshop CC 2015. After applying the profile to the image, white balance was selected for the off-white square on the X-rite colour checker board and subsequently the red-green-blue (RGB) linearity for the selected square values were set to 240 nanometres (nm) [85]. Finally, RGB values were recorded for the area surrounding the dorsal stripe, where colour sampling area included the dorsum from front legs to hind legs; flanks were excluded. Hue, saturation and lightness (HSL) values were calculated to assess the colour. Hue is an approximation of the main colour (from red to blue), while saturation measures its intensity, and lightness reflects how dark or light the colour is by quantifying the amount of black or white added to the hue. Our interpretation focused mostly on hue (H) and lightness (L). We note that while our approach allows for the unbiased extraction of colours within the human visual spectrum (300–700 nm), it ignores colours within the UV spectrum. Therefore, these colour profiles are representative for mammalian predators but the spectral sensitivity range of lizards (including *Zootoca vivipara* [86]) and birds [75, 87] is not fully covered.

From the images, the dorsal stripe was categorised by eye as either linear, reticulated or intermediate for the anterior and posterior body part. As variation between the anterior and posterior portions of the lizard’s body has previously been reported [24], dorsal patterning was recorded separately for these two areas. The dorsal pattern of each of the 394 individuals (264 females and 130 males) was categorised and dorsal colouration quantified.

### Microhabitat use

Habitat measurements were carried out in the same area where each individual was found basking and captured. The basking site was defined as the location where the lizard was first spotted and stationary. An image of the habitat was taken from above immediately after lizard capture using a Canon 70D and 60 mm lens. Each image included a 0.5 × 0.5 m quadrat square, with the basking location of approximately 0.15 × 0.15 m placed in the middle of the quadrat, and an X-rite ColorChecker board outside the quadrat. Habitat images were processed in Adobe Photoshop using the same colour-sampling methodology as for the lizard dorsal images, with profiles prepared using the X-rite ColorChecker software. The RGB values were recorded for the 0.15 × 0.15 m basking site in the centre of the quadrat. Out of all sampled individuals (N = 394), a total of 165 (120 females and 45 males) were sampled for both dorsal colouration and basking site colouration.

Habitat was categorised as made up of areas that contained grass, moss, leaf litter, angiosperm, wood, rock, bare soil, water, or ‘other’ substrate within the quadrat. Habitat category proportions were inferred using the image and a grid (25 grid squares at 3 cm x 3 cm) overlaid on the habitat image on the computer. Habitat proportions were defined as the number of grid squares of each category divided by the total number of grid squares within the quadrat. The proportion of potential cover immediately available to the lizard to escape predation was quantified as and the proportion of the quadrat which included tall grasses, wood, and other areas within the quadrat in which the individual could hide quickly. Microhabitat use could be analysed in 283 (185 females and 98 males) out of the 394 individuals.

In addition, a total of 240 random habitat images were taken throughout the study area for substrate comparison to that of basking sites. The study area can be divided into six geographical areas that are separated by dividing features, such as roads, rivers or forests [50] and random sites were determined in each of these as follows: Starting from the centre of each of the six sampling sites, a random direction was chosen by throwing a stick in the air. The stick had a designated tip, and after landing the tip of the stick determined the direction to walk. The random sampling point was reached after 20 steps, generating a nearby but separate microhabitat measure. The quadrat was then placed touching and perpendicular to the researcher’s feet and an image was taken in the same way as for the basking sites. The next random sampling point was determined by repeating the previous step, now starting from the point from which the current random sample was taken. This whole process was repeated for a total of ten random sampling points per each of the six sampling sites. Each member of the field team (N = 4) performed this step once, so that each individual sampling site contained 40 images (4 people x 10 random spots) of random habitat measures. This resulted in a total of 240 image across the whole study area (6 sampling sites x 40 images). One image was subsequently excluded due to overexposure. Habitat categories were quantified from images in the same way as for the microhabitat in which lizards were found basking.

### Statistical analyses

To assess microhabitat selection in common lizards, data on the presence, absence, or proportion of various habitat types within the proximity of lizards were modelled using Bayesian zero-and-one-inflated beta (ZOIB) regression [88]. The ZOIB distribution allows modelling of proportional data that contains both zeros and ones. In such cases the response variable, $y$, is modelled as a mixture of Bernouilli and beta distributions, from which the true zeros and ones, and the values between zero and one are generated, respectively. The probability density function is

$$f_\text{ZOIB}\left(y;\alpha,\gamma,\mu,\phi\right)=\left\{\begin{array}{lc}\alpha\left(1-\gamma\right)&y=0\\\alpha\gamma&y=1\\\left(1-\alpha\right)f\left(y;\mu,\phi\right)&0<y<1\end{array}\right.$$

where and $0<\alpha ,\gamma ,\mu <1$ and $\phi >1$. The mixture parameter, $\alpha$, determines the extent to which the Bernoulli or beta components dominate, with higher values associated indicating greater numbers of zero or one. The parameter $\gamma$ is the probability that $y=1$ when $y$ is drawn from the Bernoulli component. For a response variable where either no zeros or ones are observed $\gamma$ is fixed as either $\gamma =1$ or $\gamma =0$, respectively. Habitat categories without observations $y=1$ occurred in our data, and in these cases, the mixture parameter, $\alpha$, determines the probability that $y$ was zero rather than being a proportion modelled using the beta component. Continuous datapoints, $0<y<1$, were modelled using a beta distribution parameterised with mean $\mu$ and precision $\phi$.

For each habitat type, nine versions of the ZOIB regression model were assessed. The first null model contained single set of parameters $\alpha ,\gamma ,\mu$ and $\phi$ fitted to all lizard and random sites. The next simplest model allowed $\alpha ,\gamma ,\mu$ to vary between lizard and random sites (‘simple lizard model’). Further models allowed $\alpha ,\gamma ,\mu$ to also vary between female and male lizards, between oviparous and viviparous, and finally by both sex and parity mode. Each model other than the null was implemented with a single $\phi$ parameter shared by all observations and alternatively with two $\phi$ parameters for lizard and random sites. Model comparison was done through comparison of deviance information criterion (DIC) [89], with the best model identified as that with the lowest DIC, unless a simpler model was found with a DIC score differing by less than two, in which case the simpler model was preferred.

Bayesian models were implemented with minimally informative priors for each observation class, where class may represent either random or common lizard sites in simpler models or may represent sites with common lizards of a particular sex or parity mode in more complex models. For each observation class, $j$, prior distributions were: for the mixture parameter $\text{l}\text{o}\text{g}\text{i}\text{t}\left({\alpha }_{j}\right)={\alpha `}_{j} \text{w}\text{h}\text{e}\text{r}\text{e}\,{\alpha `}_{j}\sim\text{N}\text{o}\text{r}\text{m}\text{a}\text{l}\left(0, 1000\right)$; for the probability in the logistic regression probability $\text{l}\text{o}\text{g}\text{i}\text{t}\left({\gamma }_{j}\right)={\gamma `}_{j} \text{w}\text{h}\text{e}\text{r}\text{e}\,{\gamma `}_{j}\sim\text{N}\text{o}\text{r}\text{m}\text{a}\text{l}\left(0, 1\right)$; for the beta regression mean $\text{l}\text{o}\text{g}\text{i}\text{t}\left({\mu }_{j}\right)={\mu `}_{j} \text{w}\text{h}\text{e}\text{r}\text{e}\,{\mu `}_{j}\sim\text{N}\text{o}\text{r}\text{m}\text{a}\text{l}\left(0, 1000\right)$; and beta regression precision ${\phi }_{j}={e}^{{\phi `}_{j}} \text{w}\text{h}\text{e}\text{r}\text{e}\,{\phi `}_{j}\sim\text{N}\text{o}\text{r}\text{m}\text{a}\text{l}\left(0, 100\right)$. Models were specified in the JAGS language and run using JAGS v4.3.0 [90] initiated from *R* (R Core Team 2013) using the package runjags, with DIC calculated using the function *extract.jags* [91].

Next, we assessed if common lizard dorsal colouration was dependent on any attributes of the individual (e.g., parity mode, sex, length, weight) and if it matched their background colouration. We extracted HSL (hue, saturation, lightness) values from the images of dorsal colouration, basking site, and habitat. Next, a generalised linear model (GLM) was used to identify if average dorsal colouration (measured as HSL) correlated with parity mode, sex, body length, and weight of an individual (model 1). Similarly, a GLM with a binomial distribution was used to establish if dorsal patterning (reticulated, intermediate or linear) depended on parity mode, sex, body length and weight (model 2). Using an ANOVA, we tested if dorsal colouration showed a correlation with dorsal patterning (model 3). Next, we checked if colouration of the whole microhabitat correlated with the basking site colouration (model 4). We then tested if average dorsal colouration was associated with basking site and sex (model 5), and with the colouration of the whole microhabitat and sex (model 6). Consequently, we also tested the same for dorsal patterning (model 7 and 8).

### Supplementary Information

**Additional file 1: Table S1.** Parameters in zero-and-one-inflated beta regression models favoured by DIC.

**Additional file 2: Table S2.** Statistical models performed on lizard colouration and patterning. The effect of any significantly associated variable in a model is shown.

**Additional file 3: Figure S1.** Dorsal colouration differs between parity modes and correlates with body weight. Viviparous (A) and larger (B) individuals tended to have larger hue values compared to oviparous and smaller individuals.

**Additional file 4: Figure S2.** Histograms of A) the wider habitat lightness and B) basking site lightness.

## Data Availability

Data are available from the Univ. of Glasgow Enlighten Repository 10.5525/gla.researchdata.1417. Bayesian models specified in JAGS syntax and R code required to run them on an example dataset are available at https://github.com/will-harvey/JAGSeco-evo.
