# Supplementary material for: Common lizard microhabitat selection varies by sex, parity mode, and colouration
Source: BMC Ecol Evol. 2023 Sep 4;23:47. doi: 10.1186/s12862-023-02158-2 (PMC10478496; doi:10.1186/s12862-023-02158-2)
Supplement: Supplementary file 1 — Additional file 1: Table S1. Parameters in zero-and-one-inflated beta regression models favoured by DIC. [file 12862_2023_2158_MOESM1_ESM.pdf]

Additional file 1. Table S1. Parameters in zero-and-one-inflated beta regression models favoured by DIC.

Habitat type	Class	Model parameters			
		Mixture parameter (α)[1]	Bernoulli distribution probability (γ)[2]	Beta distribution mean (μ)	Beta distribution precision (ϕ)
Angiosperm	Random	0.59	0	0.03	37.90
	Lizard, oviparous	0.87	0	0.14	11.15
	Lizard, viviparous	0.65	0	0.11	11.15
Grass	Random	0.04	0.67	0.70	2.71
	Lizard	0.04	0.74	0.55	2.71
Leaf litter	Random	0.90	0	0.1	29.61
	Lizard	0.93	0	0.18	8.55
Moss	Random	0.88	0	0.24	3.97
	Lizard, oviparous	0.71	0	0.22	3.97
	Lizard, viviparous	0.86	0	0.19	3.97
Rock	Random	0.83	0	0.29	2.05
	Lizard	0.89	0	0.27	6.04
Water	Random	0.99	0	0.14	11.92
	Lizard, oviparous	0.91	0	0.19	11.92
	Lizard, viviparous	0.99	0	0.14	11.92
Wood	Random	0.86	0	0.22	3.14
	Lizard, female	0.42	0	0.47	3.14
	Lizard, male	0.47	0	0.42	3.14
Other	Random	0.94	0	0.14	8.89
	Lizard, oviparous	0.97	0	0.39	8.89
	Lizard, viviparous	0.92	0	0.19	8.89
Cover	Random	0.06	0.66	0.65	2.31
	Lizard, oviparous	0.01	0.61	0.54	2.31
	Lizard, viviparous	0.12	0.59	0.56	2.31
Bare ground	Random	0.58	0	0.18	4.91
	Lizard, female	0.57	0	0.16	10.07
	Lizard, male	0.67	0	0.19	10.07

[1]Where $\gamma = 0$, the mixture parameter, α, represents the probability of the microhabitat being absent (proportion = 0).
[2]Where $\gamma = 0$, no microhabitat proportions of 1 where observed, otherwise γ is the estimated probability that a discrete proportion is 1 rather than 0.
