# Supplementary material for: Common lizard microhabitat selection varies by sex, parity mode, and colouration
Source: BMC Ecol Evol. 2023 Sep 4;23:47. doi: 10.1186/s12862-023-02158-2 (PMC10478496; doi:10.1186/s12862-023-02158-2)
Supplement: Supplementary file 2 — Additional file 2: Table S2. Statistical models performed on lizard colouration and patterning. The effect of any significantly associated variable in a model is shown. [file 12862_2023_2158_MOESM2_ESM.pdf]

Additional file 2. Table S2. Statistical models performed on lizard colouration and patterning. The effect of any significantly associated variable in a model is shown.

model ID	response variable	explanatory variables	test	sign. associated variables	num. df	den. Df	t - or F - value	p - value	
1	dorsal colouration (H)	sex, body length, body weight	GLM	body length	2	301	4.61	<0.001	***
	dorsal colouration (S)	sex, body length, body weight	GLM	sex	4	299	2.29	<0.05	*
				body length			3.49	<0.01	**
				body weight			-3.44	<0.001	***
	dorsal colouration (L)	sex, body length, body weight	GLM	-	-	-	-	-	
2	dorsal patterning	sex, body length, body weight	GLM	sex	3	387	3.051	<0.01	**
				body length			2.785	<0.01	**
3	dorsal colouration (H)	dorsal patterning	ANOVA	dorsal patterning	2	299	3.04	<0.05	*
	dorsal colouration (S)	dorsal patterning	ANOVA	-	-	-	-	-	
	dorsal colouration (L)	dorsal patterning	ANOVA	dorsal patterning	2	299	6.08	<0.01	**
4	basking site colouration (H)	habitat colouration (H)	LM	habitat colouration (H)	2	155	8.747	<0.001	***
	basking site colouration (S)	habitat colouration (S)	LM	habitat colouration (S)	2	155	11.35	<0.001	***
	basking site colouration (L)	habitat colouration (L)	LM	habitat colouration (L)	2	155	11.51	<0.001	***
5	dorsal colouration (H)	basking site colouration (H), sex	GLM	basking site colouration (H)	2	162	3.567	<0.001	***
	dorsal colouration (S)	basking site colouration (S), sex	GLM	-	-	-	-	-	
	dorsal colouration (L)	basking site colouration (L), sex	GLM	basking site colouration (L)	2	160	-2.139	<0.05	*
6	dorsal colouration (H)	habitat colouration (H), sex	GLM	habitat colouration	2	157	5.293	<0.001	***
	dorsal colouration (S)	habitat colouration (S), sex	GLM	-	-	-	-	-	
	dorsal colouration (L)	habitat colouration (L), sex	GLM	-	-	-	-	-	
7	dorsal patterning	basking site colouration (H, S, L), sex	GLM	-	-	-	-	-	
8	dorsal patterning	habitat colouration (H, S, L), sex	GLM	-	-	-	-	-	

Abbreviations: H = hue, S = saturation, L = lightness.
