# Supplementary material for: Common lizard microhabitat selection varies by sex, parity mode, and colouration
Source: BMC Ecol Evol. 2023 Sep 4;23:47. doi: 10.1186/s12862-023-02158-2 (PMC10478496; doi:10.1186/s12862-023-02158-2)

Additional file 3 - Figure S1. Dorsal colouration differs between parity modes and correlates with body weight. Viviparous (A) and larger (B) individuals tended to have larger hue values compared to oviparous and smaller individuals.

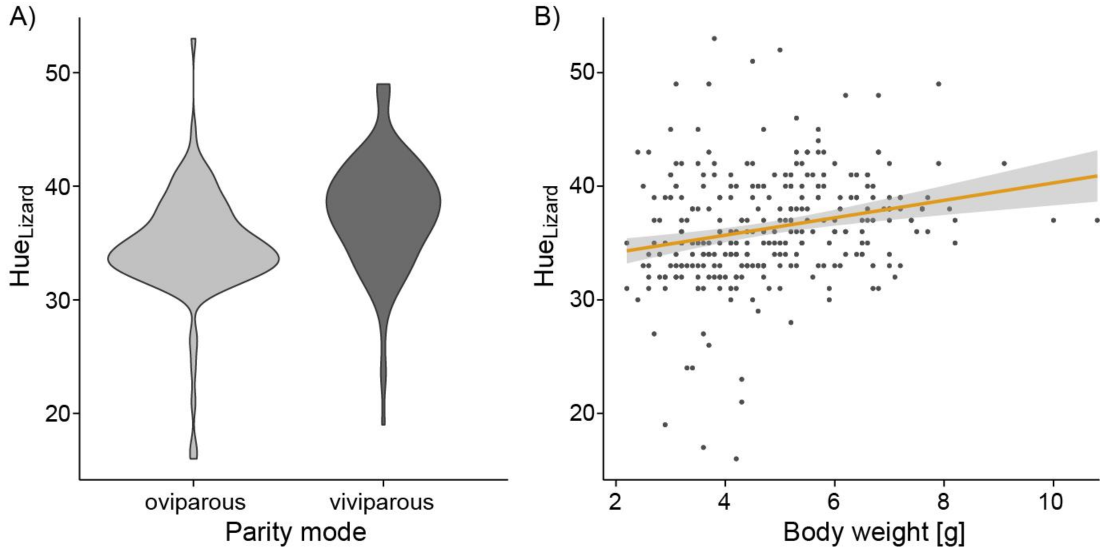

Supplement: Supplementary file 3 — Additional file 3: Figure S1. Dorsal colouration differs between parity modes and correlates with body weight. Viviparous (A) and larger (B) individuals tended to have larger hue values compared to oviparous and smaller individuals. [file 12862_2023_2158_MOESM3_ESM.pdf]
