# Supplementary material for: Common lizard microhabitat selection varies by sex, parity mode, and colouration
Source: BMC Ecol Evol. 2023 Sep 4;23:47. doi: 10.1186/s12862-023-02158-2 (PMC10478496; doi:10.1186/s12862-023-02158-2)

Additional File 4. Figure S2. Histograms of A) the wider habitat lightness and B) basking site lightness.

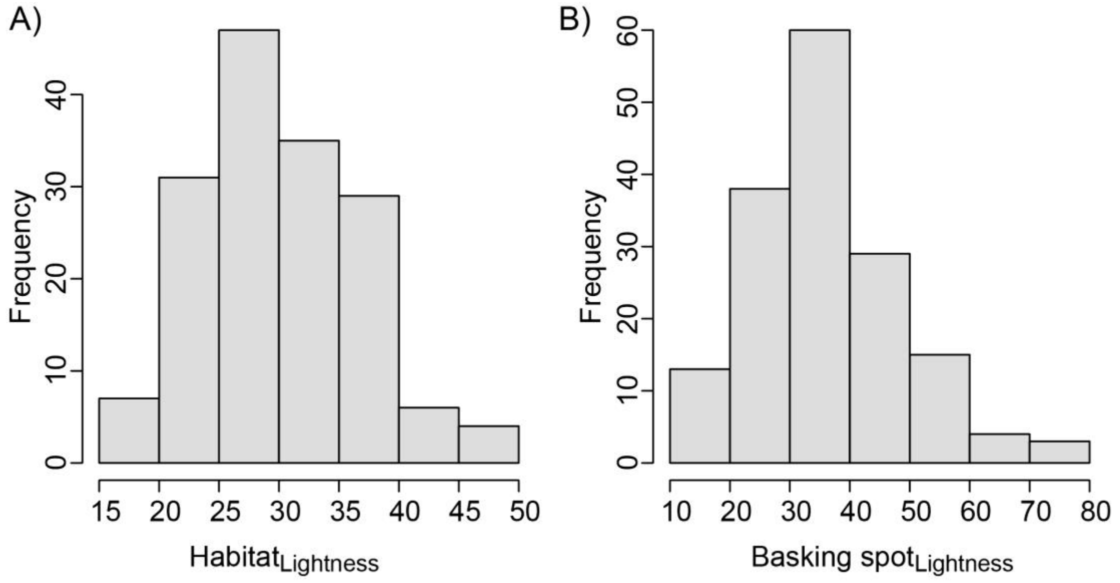

Supplement: Supplementary file 4 — Additional file 4: Figure S2. Histograms of A) the wider habitat lightness and B) basking site lightness. [file 12862_2023_2158_MOESM4_ESM.pdf]
